# The assessment of a structured online formative assessment program: a randomised controlled trial

**DOI:** 10.1186/1472-6920-14-8

**Published:** 2014-01-09

**Authors:** Edward Palmer, Peter Devitt

**Affiliations:** 1 School of Education, University of Adelaide, Adelaide, Australia; 2 School of Medicine, University of Adelaide, Adelaide, Australia

**Keywords:** Formative assessment, Online learning, Summative assessment, Engagement

## Abstract

**Background:**

Online formative assessment continues to be an important area of research and methods which actively engage the learner and provide useful learning outcomes are of particular interest. This study reports on the outcomes of a two year study of medical students using formative assessment tools.

**Method:**

The study was conducted over two consecutive years using two different strategies for engaging students. The Year 1 strategy involved voluntary use of the formative assessment tool by 129 students. In Year 2, a second cohort of 130 students was encouraged to complete the formative assessment by incorporating summative assessment elements into it. Outcomes from pre and post testing students around the formative assessment intervention were used as measures of learning. To compare improvement scores between the two years a two-way Analysis of Variance (ANOVA) model was fitted to the data.

**Results:**

The ANOVA model showed that there was a significant difference in improvement scores between students in the two years (mean improvement percentage 19% vs. 38.5%, p < 0.0001). Students were more likely to complete formative assessment items if they had a summative component. In Year 2, the time spent using the formative assessment tool had no impact on student improvement, nor did the number of assessment items completed.

**Conclusion:**

The online medium is a valuable learning resource, capable of providing timely formative feedback and stimulating student-centered learning. However the production of quality content is a time-consuming task and careful consideration must be given to the strategies employed to ensure its efficacy. Course designers should consider the potential positive impact summative components to formative assessment may have on student engagement and outcomes.

## Background

Formative assessment is designed to inform both student and teacher about the progress of the student and may be described as ‘assessment *for* learning’ [1]. It may manifest itself in many of the same formats as summative assessments, but the timing and intent is vastly different. Whereas summative assessment seeks to ensure that students cannot proceed with their study or profession without passing the assessment, formative assessments are designed to help students attain the knowledge, understanding and skills crucial to their subject and course. Student use of formative assessment exercises is intended to create awareness of their own weaknesses in order to formulate plans to address them. When well designed, a formative assessment process should reduce students’ dependence on the teacher but, through good feedback, the teacher will still play an important role. A recurring complaint from many students is the paucity and usefulness of feedback [2-4], and their desire for more learning materials. It can be a challenge for educators to meet these requests due to lack of time and resources and the growing number of students. It would assist educators if they had an efficient means of providing feedback on a large scale that was meaningful to the student and aided their learning. Online learning provides such a method, but many educators are unsure of the technology and the potential outcomes.

In many medical curricula much of the teaching is delivered by clinicians whose primary focus is patient care and not student education [5]. The role of the clinician educator is challenging and can be poorly supported [6,7] and whilst many may express interest in providing formative assessment, time constraints are likely to impact on the ability to provide comprehensive, consistent tasks for students to learn from and provide feedback to complement the learning. When measured in terms of a clinician’s time, production of formative assessment materials for the online medium is an expensive exercise. If time, effort and money are to be spent in this direction, evidence must be provided of the benefits and cost-effectiveness.

Meta analyses of the value of computers in education provide conflicting results. A meta-analysis of studies comparing distance education tools with classroom instruction showed wide variability and almost no effect size [8]. The authors reported findings suggesting that the use of a problem-based learning strategy was a good predictor of achievement and of positive attitudes towards distance learning, but overall, they found the results so varied and inconsistent that they felt unable to make strong recommendations about appropriate distance education practices to provide good learning outcomes. A meta-analysis in orthodontics showed minimal benefit for online learning whilst raising the issues of cost and time effectiveness [9]. A meta-analysis of 47 comparative studies carried out in 1992 [10] showed a significant positive result for computer-assisted instruction over traditional methods, with a large effect size of 0.41. A similar conclusion was supported by later studies [11-15].

A US study [15], based on a study of thousands of articles on online education from 1996–2008, suggested that instruction combining online and face to face elements had improved outcomes for students and in today’s learning environment students might be expected to look to the online medium for tools to enhance their learning experience. Contrary to such expectations students do not always take advantage of these opportunities. We have previously shown that clinical medical students made little use of online formative assessment materials provided informally even though those very same students judged the resource to be of practical value and useful in their own studies [16]. The poor uptake was reflected in scant educational gain as measured in terms of any improvement in cognitive skills in multiple-choice tests. Similar studies have indicated different outcomes [17], so there is a clear need to determine what strategies can be effective to encourage students to study and to learn online.

We have undertaken a study to observe and measure the educational worth of online formative assessment and the effectiveness of two different strategies to encourage its use for learning.

## Methods

This study had two objectives, namely to observe and quantify the use of specifically designed online materials and to compare two strategies for encouraging the use of the learning material by students by looking at learning outcomes and usage patterns.

The online materials were case based scenarios and they were delivered using the program Medici (http://www.emedici.com) [16]. Medici presents a clinical case in stages, providing information and a question to the user at each stage. The question can be answered by selecting one or many of a series of choices (like a multiple choice question) or by providing a short answer. In all cases the student is provided with feedback instantly regardless of whether they have selected the wrong or right answer. The feedback is provided as a model answer for short answer questions and as comprehensive, tailored feedback for each choice made by the student in the multiple choice format. Students are able to view video or still images and at the end of the case they are provided with a summary of the salient points in the case including contentious management decisions and further reading as required.

All students (129 in Year 1 and 130 in Year 2) enrolled in the surgical home unit component of the MBBS curriculum participated in the study. Prior to the start of the academic year, as part of Faculty operations, students were randomly allocated to one of four groups with stratification for gender, international status and academic ability. International status refers to the recruitment of overseas students into the program. Academic ability is based on previous year’s examination results. Each group of students had a nine-week attachment to a surgical clinic.

The study was conducted over two consecutive years using two different strategies for engaging students. In the first year [16], 12 clinical scenarios, produced by a senior practicing surgeon, were made available to the students. These were available for use throughout a nine week surgical attachment but contained no assessable elements. Students were pre and post tested using a 46 question multiple choice exam. The exam was identical for both pre and post test and security of the questions was maintained by ensuring that the students were held in a closely invigilated room whilst they completed the exam. No access to the exam was allowed for students outside of the testing room and no copies of the exam were permitted. Students were informed repeatedly that the content of the online clinical scenarios would assist them greatly in the exam. The exam questions were based on the material in the clinical scenarios but were substantially different so that rote learning of content in the online material would be of no assistance.

**Figure 1:**
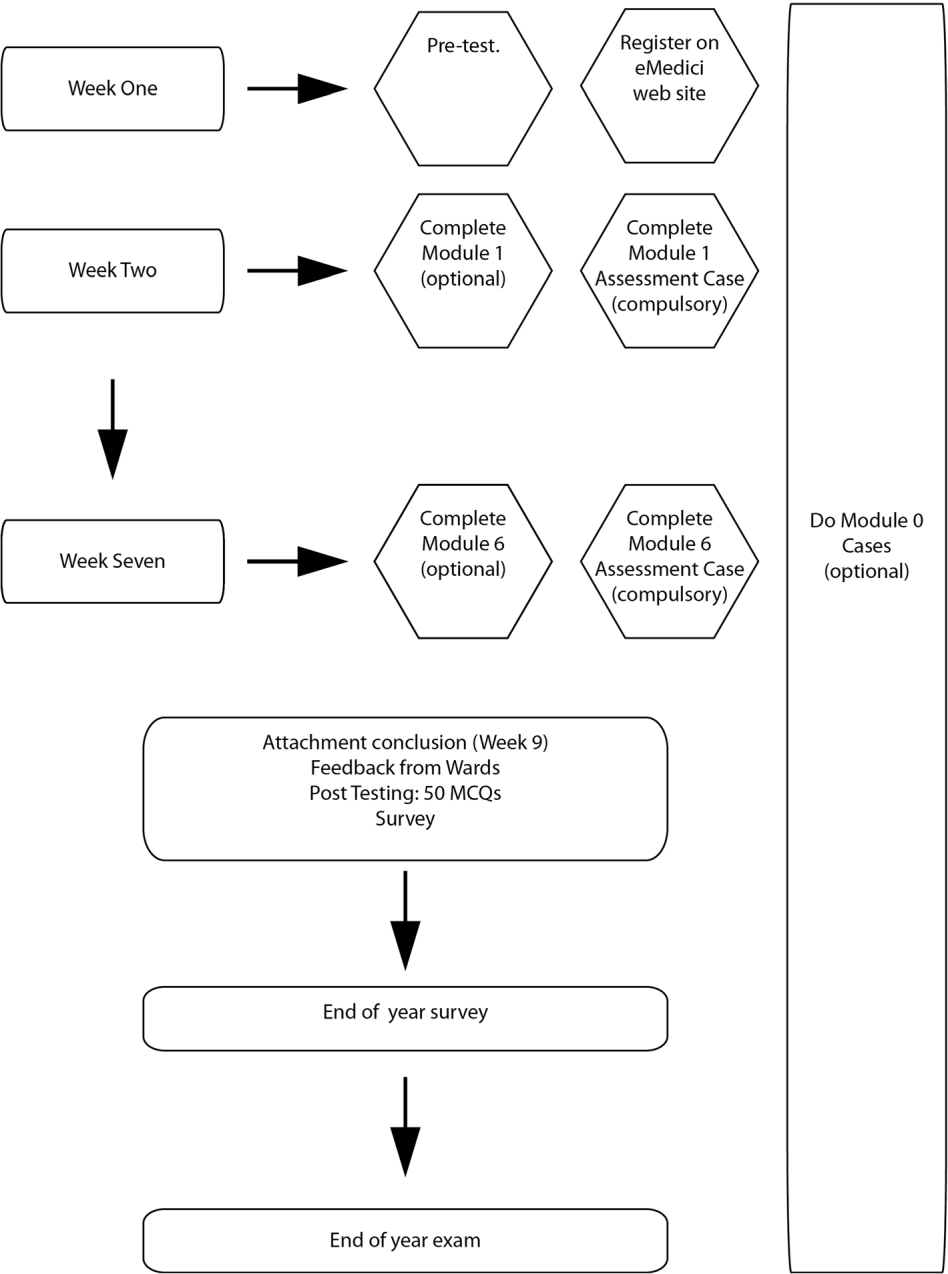
Course structure including eAssessments.

In Year 2 of the study, 38 new scenarios were constructed. These covered key areas of the core curriculum in surgery and at least six different scenarios were made available on a weekly basis for six weeks of the nine-week attachment (Figure 1). Whereas in the first year of the study no inducement was made to use the material other than encouragement, the material in the second year of the study contained a component, which was summatively assessed, and the material was delivered using a different structure. Each block of clinical scenarios in Year 2 was accompanied by a short test of 10 Multiple Choice Questions (MCQs). The MCQs were based on material discussed in the clinical scenarios. Study of the clinical scenarios was optional but the weekly tests were mandatory. At the start of each attachment student were briefed on the availability of this online learning resource and the need to complete the tests – which counted towards their overall assessment. No restrictions were made as to how or when each test should be completed except that it had to be completed during the week it became available.

The MCQ tests were combined and counted for 10% of the grade allocated to students in the surgical attachment. A passing mark could easily be obtained from the assessment case if the other cases in the module were completed. They contained little feedback and were designed for summative purposes only. A further 18 clinical scenarios were available for use at anytime (Module 0), but the assessment modules were released weekly. The last module was due to be completed at least 2 weeks before the end of attachment summative assessment. Overall, students had 62 cases to study with, although only 6 were compulsory (the assessment cases).

The aims of structuring the formative assessment material in this way were to ensure that:

• Students did not feel unduly pressured about other deadlines during the formative assessment period. The two-week gap before the end of attachment examination was designed so that students would focus only on one assessment at a time.

• Students could feel confident in their ability to complete the weekly assessment provided they had studied the accompanying clinical scenarios in that week’s module.

• The time required to work on the formative assessment exercises was not excessive. Six cases a week were anticipated to take 60–90 minutes to complete.

The cases used for this formative assessment process were carefully selected and constructed in the knowledge that the students who used them would be working in different environments and with different clinical materials. For example, students attached to the colorectal unit at one of the tertiary referral centres would not see the breadth of clinical material available to a student working in one of the rural settings, but might be able to work in more of a collaborative environment than the more isolated rural practices. Thus cases were produced to reflect the broad scope of surgical practice and to encourage the user to study further in the designated areas.

No restrictions were made as to how the formative assessment material was to be used. If students chose to collaborate on the cases, that was deemed to be a positive outcome. The goal of the material was to expose students to material they may not see on wards and to engage in the diagnostic and management issues in a constructive and non-threatening manner. Group participation in achieving this was not considered to be a negative result, especially for students who may be isolated in small groups in rural communities. It was acknowledged that at some stage students were likely to also collaborate on the assessment case provided each week, potentially making the assessment non-discriminatory. This risk was balanced against the potential benefit of students completing the formative material, which was the main goal of creating the material.

The time spent on the cases was monitored by the online system. Where the system showed that student had been active for longer than an hour checks were made to ensure that activity had been occurring during that time otherwise that data was excluded.

The study was formally evaluated by pre- and post-testing of cognitive skills. The structure of the 9-week surgical attachment was identical in both years except that the assessment changed slightly. The second phase of the project did not include MEQs as part of the assessment, because independent of this study we had shown that well constructed MCQs were capable of testing the higher order cognitive skills of reasoning and judgement and did so more reliably than MEQs [18]. The pre and post tests used in Year 2 were identical to those used in Year 1 and were administered in identical circumstances. An example of a type of question is provided below.

A 58-year-old man presents with a three month history of dysphagia on a background of gastro-esophageal reflux, the latter which has been present for many years. An endoscopy shows a well-defined ulcer with raised margins and surrounding inflammation extending up to the mid-oesophagus. The ulcerated area is biopsied and shows columnar-lined epithelium with areas of high-grade dysplasia.

Which one of the following is the most appropriate next step in management?

• *Repeat the endoscopy and biopsies*

• *Perform manometric and pH studies*

• *Start on a proton pump inhibitor and repeat the endoscopy in three months*

• *Perform a barium contrast study of the oesophagus*

• *Perform argon ablation therapy of the inflamed tissue*

The end of attachment assessment was delivered fully online. There were four groups, labelled A to D, who completed their surgical attachment in sequence (Group A at the beginning of the year and Group D at the end). Note that group A was not provided a pre-test due to scheduling difficulties. Group A also had slightly fewer cases available for study (55 as opposed to 62 for other groups), as they were still under development.

The results of the pre-and post-tests were compared with the equivalent data obtained for the previous year when there were few clinical scenarios and no obligation to study the material [16].

To compare improvement scores between the two years while adjusting for group, a two-way Analysis of Variance (ANOVA) model was fitted to the data. In the model, the improvement score (difference between the post-test and pre-test result) was entered as the outcome variable, while year and Group (A, B, C or D) were entered as predictor variables. Note that Group A in the second year was not included in this analysis as the improvement scores were undefined.

Given that improvement scores were undefined in Group A in the second year (as they had missing pre-test scores), a second set of analyses comparing only post-test results was conducted. To compare post-test scores between the two years while adjusting for group, a two-way ANOVA model was fitted to the data. In the model the post-test score was entered as the outcome variable, while year and Group (A, B, C or D) were entered as the predictor variables.

To compare the percentage of support cases (Modules 1–6) completed with the percentage of incidental (Module 0) cases completed a negative binomial generalised estimating equation (GEE) model was fitted to the data. In the model, the number of cases completed was entered as the outcome variable, while type of case (support/incident), Group (A, B, C or D) and the interaction between type of case and group were entered as predictor variables. The total number of cases available was included as an offset variable to account for the slightly fewer cases available to Group A.

To compare the time spent online between the four groups in year 2, a one-way ANOVA model was fitted to the data. Time spent online by students was entered as the outcome variable in the model, while Group (A, B, C or D) was entered as the predictor variable.

## Results

The raw percentages for the pre- and post-test are provided as Table 1. All groups in Year 2 obtained a higher score in the end of attachment assessment than the comparable group in Year 1. The pre-test scores were similar, but group C in Year 2 performed significantly worse than the equivalent group in Year 1. In Year 1 the pre to post-test improvement ranged from 40-66%. In Year 2 the improvement ranged from 85 to 120%. Group D in Year 2 performed better than any of the other 3 groups.

**Table 1:** Test results for Groups A-D over two years

**Year 1**	**MCQ pre-test**	**MCQ postest**	**Percent improvement**
**A (n = 30)**	**35 ± 2**	**58 ± 1**	**66%**
**B (n = 33)**	**42 ± 1**	**59 ± 2**	**40%**
**C (n = 33)**	**47 ± 2**	**66 ± 2**	**40%**
**D (n = 33)**	**45 ± 1**	**63 ± 1**	**37%**
**Year 2**	**MCQ pre-test percentage**	**MCQ postest**	**Percent improvement**
**A (n = 33)**	**N/A**	**75 ± 2**	**N/A**
**B (n = 34)**	**43 ± 2**	**76 ± 1**	**77%**
**C (n = 32)**	**35 ± 2**	**76 ± 2**	**117%**
**D (n = 31)**	**39 ± 3**	**83 ± 2**	**112%**

Marks are given as score (percent) ± standard error.

The ANOVA model showed that after adjusting for group there was a significant difference in improvement scores between students in the two years (mean improvement 8.8 marks (19%) vs. 17.9 marks (38.5%), p < 0.0001). The model showed that after adjusting for group there was also a significant difference in post-test scores between students in the two years (mean score 28.3 marks (61%) vs. 35.7 marks (78%), p = 0.0004).

The patterns of usage of the online material for Year 2 are summarised in Table 2. Apart from the optional Module ‘0’, which contained 18 clinical scenarios, the behaviour in the groups was similar. Most students attempted and completed cases in each module (overall 90% completion rate). There was often more than one attempt on each case, where the overall completion rate fell from near 90% to 65%. Module 0 had fewer students attempting cases and lower completion rates for the cases that were attempted. When module 0 is compared with all other modules (Table 2), it is clear that fewer cases were completed from this optional module.

**Table 2:** Patterns of usage for Modules 0 and 1-6 (Year 2)

**Group**	**Module**	**Av. no. students attempting cases in this module**	**Av. no. students completing cases in this module**	**% complete**	**Av. no. attempts at cases in this module**	**Av. no. attempts completed for the cases in this module**	**% complete**
**A**	**0**	**18 ± 1**	**15 ± 1**	**80 ± 3**	**42 ± 6**	**19 ± 2**	**49 ± 4**
**B**	**0**	**26.6 ± 0.8**	**22 ± 1**	**83 ± 2**	**64 ± 4**	**32 ± 2**	**51 ± 3**
**C**	**0**	**14 ± 1**	**11 ± 1**	**77 ± 5**	**24 ± 3**	**13 ± 2**	**57 ± 5**
**D**	**0**	**9.9 ± 0.8**	**7.6 ± 0.8**	**74 ± 3**	**16 ± 2**	**9 ± 1**	**52 ± 4**
		**17.1 ± 0.9**	**13.8 ± 0.9**	**78 ± 2**	**36 ± 3**	**18 ± 2**	**52 ± 2**
**A**	**1–6**	**25.7 ± 0.7**	**23.0 ± 0.8**	**87 ± 3**	**59 ± 4**	**36 ± 2**	**65 ± 2**
**B**	**1–6**	**30.9 ± 03**	**28.1 ± 0.5**	**91 ± 1**	**92 ± 5**	**55 ± 2**	**62 ± 2**
**C**	**1–6**	**25.4 ± 0.4**	**23.8 ± 0.5**	**94 ± 1**	**63 ± 3**	**39 ± 1**	**66 ± 2**
**D**	**1–6**	**21.8 ± 0.6**	**19.0 ± 0.6**	**87 ± 2**	**46 ± 2**	**28 ± 2**	**62 ± 2**
		**25.9 ± 0.4**	**23.5 ± 0.4**	**89.5 ± 0.9**	**65 ± 2**	**40 ± 1**	**64 ± 1**

Statistical data presented as average ± standard error.

When the percentage of support cases (Modules 1–6) completed was compared with the percentage of incidental (Module 0) case completed, the interaction term was statistically significant (Table 3). The interaction term showed that the difference between the proportion of support and incident cases completed varied across the four groups (p = 0.035). The proportion of completed cases was higher for support cases than incidental cases in all groups, particularly in Group D. In all groups, the proportion of completed incidental cases was found to be lower than the proportion of completed support cases (p < 0.05). The column labelled rate ratio (Table 3) expresses the ratio of the two proportions. The proportion of completed incidental to support cases was highest in Group B (rate ratio = 0.859) and lowest in Group D (rate ratio = 0.554).

**Table 3:** Differences of adjusted means

**Effect**	**Group**	**Type of case**	**_Group**	**_ Type of case**	**Estimate**	**Standard error**	**DF**	**Chi-square**	**P-value**	**Rate ratio**
Type of Case*Group	A	Incident	A	Support	-0.3008	0.1072	1	7.87	0.0050	0.740
Type of Case*Group	B	Incident	B	Support	-0.1525	0.0607	1	6.31	0.0120	0.859
Type of Case*Group	C	Incident	C	Support	-0.4058	0.1169	1	12.05	0.0005	0.666
Type of Case*Group	D	Incident	D	Support	-0.5901	0.1691	1	12.17	0.0005	0.554

Within Modules 1 to 6, students did not devote their time solely to the summative assessment cases (the minitests of 10 MCQs). Regardless of group or module, the cases provided for self-directed learning were often attempted and completed. Most students were sufficiently interested to move through the scenarios with only 7% attempting a case failing to move beyond the first screen (Table 4). Students in group D spent less time, attempted and completed significantly fewer cases than other groups. Group B behaved in an opposing manner, attempting and completing more cases than other groups.

**Table 4:** Percentages of students failing to proceed with a case after the first screen

**Group**	**Left after 1**^**st **^**screen (percentage of all attempts)**
**A**	194 (6%)
**B**	391 (8%)
**C**	234 (7%)
**D**	175 (8%)

Year 2 students attempted more cases (Tables 5 and 6) compared with Year 1. In the optional module alone, twice as many students attempted cases on average than in Year 1, where all cases were optional. In the modules where the minitest was present, the number of students attempting cases was approximately 3 times higher. Students spent between 10 and 20 hours on the Medici cases. When considering the extra cases involved, this is comparable to the best performing group from Year 1 (Group C) which spent an average of 2 hours on case studies using Medici.

**Table 5:** **Student usage of the** ***Medici*** **interactive online resource from beginning of attachment to the end of the academic year for Year 1**

**Group**	**No students attempting cases (total no. students)**	**Median number of cases attempted**	**Median number of screens (median 10 screens per case)**	**Median time spent (minutes)**
A	4 (34)	2 [1.5–5]	40 [12.5–63]	28 [9–30]
B	2 (33)	3.5 [3,4]	37 [14–60]	26 [9–42]
C	11 (28)	11 [6–12]	129 [59–240]	90 [20–143]
D	6 (30)	7 [2–10.5]	82.5 [17–152]	53 [25–140]

Results are expressed as median [interquartile range].

**Table 6:** **Student usage of the** ***Medici*** **interactive online resource from beginning of attachment to the end of the academic year for Year 2**

**Group**	**No students attempting cases (total no. students)**	**Median number of cases attempted**	**Median number of screens (median 10 screens per case)**	**Median time spent (minutes)**
A	33 (33)	44 [29.5-54]	410 [230–560]	506 [340–890]
B	34 (34)	57.5 [48.25-60]	560 [450–800]	1045 [670–1510]
C*	30 (32)	46.5 [41.75-56.25]	450 [350–580]	765 [490–1060]
D	29 (31)	42 [35–51]	360 [230–440]	560 [370–770]

Results are expressed as median [interquartile range].

*Two students from Group C dropped out of the course.

The ANOVA model demonstrated that there was a significant difference between the four groups from Year 2 in the time spent online (p < 0.0001). The mean time spent online by students was highest in Group B (median = 1045 minutes) and lowest in Group D (median = 560 minutes).

The post hoc tests revealed that students in group B spent more time online than students in Groups A, C and D (p <0.0001, p = 0.007 and p <0.0001 respectively) and attempted significantly more cases than the same groups. No significant differences were found between Groups A, C and D.

To test whether improvement scores were related to Group (B, C or D), total time spent online, number of support cases completed or number of incident cases completed, a linear regression model was fitted to the data. Only the student group was found to be a significant predictor of improvement scores (p = 0.046). There was no evidence that the number of incident cases completed, the number of support cases completed or time spent online had an influence on improvement scores. Students in Group B were found to have significantly lower improvement scores than students in Group C (15.3 marks (7%) vs. 18.9 marks (8.7%), p = 0.023).

To test whether post-test scores were related to Group (A, B, C or D), total time spent online, number of support cases completed or number of incident cases completed, a linear regression model was fitted to the data. Both group (p = 0.0012) and the number of support cases completed (p = 0.013) were found to be predictive of post-test scores. For every single unit increase in the number of support cases completed, the post-test score increased by an average of 0.13 units (p = 0.013). Group D was found to have higher post-test scores than Groups A, B and C (p = 0.0003, p = 0.001 and p = 0.002 respectively).

## Discussion

It is apparent that in every outcome measured in this study, the fourth year group in Year 2 improved on the outcomes of the previous year’s group. They made greater use of the online material and performed better in the end-of-attachment exam. It is worth noting that there were many differences from Year 1 to Year 2. The content was expanded allowing the students more cases to elect to complete, there was an assessment component to the Year 2 cohort and the delivery method was quite different for Year 2. It is also possible that students discussed the pre and post tests thus introducing potential bias. It is tempting to conclude that the greater use of online material caused the improvement in learning outcomes as measured in this study, especially as the comparison group in year 1 was in many respects identical to the group in Year 2, but without the structured formative assessment strategy.

What is probably more important is to consider how these differences came about. The exam material addressed the learning objectives of the cases, but did not mirror it, so rote learning is not a reasonable conclusion to draw. It is certainly possible that the cases themselves taught the students all they needed to do well in the exams, but that may be downplaying the effect of the learning tool. It could be that the use of the cases inspired students to seek more information in related fields, visit a greater number of patients or examine old case notes. It is also likely that they would have discussed management decisions suggested within the case scenarios with clinicians, especially if they disagreed with some of those decisions. It is worth remembering that there were not necessarily any correct answers in many cases, only decisions based on evidence and clinical acumen. Different clinicians are likely to make different, but not incorrect decisions about how a patient is managed. Learning that these differences exist and the reasons behind them may help students build up a databank of knowledge, perhaps like the scripts suggested by those proponents of script concordance methods of learning and assessing [19].

An observation of interest is that time spent on the online cases was not a strong predictor of post-test outcome. It might be supposed that the more work the better the outcome, but it is worth examining the time spent on the cases. The interquartile range for group A was 340–890 minutes and for group D it was 370–770 minutes. These are non-trivial times. Six hours for the student represented by the lowest bound in these ranges is a significant amount of study time on a formative exercise. It is possible that the student achieved all that could be gained from the exercises by spending that amount of time on them. In other words, it is possible that there is a threshold where nothing further can be gained by repeating case scenarios. Intuitively this makes sense. There comes a point where there is a need to learn in a different way or to recognise that what something has to offer has been learned to an appropriate level. If that is the case then students that spent twice this time studying formative cases may have not been using their time effectively. Perhaps it is important to tell students not to study too hard using one type of learning?

The behaviour of different groups was interesting. Groups B and C dedicated more time to the Medici cases than groups A and D, with Group B spending significantly more time than the other groups. The reason behind this is likely due to acclimatisation and examinations. Group A was the first group to do the cases and the surgical attachment was their first clinical attachment. In this situation, the students would not have the benefit of advice from other students in their cohort about the value of tasks and they would have been spending significant amounts of time familiarising themselves with hospital structures as well as the appropriate protocols when dealing with patients, interns and senior clinicians. This process takes significant time and may well have taken it away from formative assessment exercises. Group D was the last attachment and although they would have understood the requirements of an attachment at that stage, they would have also been focusing on the general end-of-year examinations and preparing for them. In this instance, the formative assessments cases, although useful for one discipline, were not going to be of assistance in others and thus were perhaps given a lower priority. Groups B and C were likely the most relaxed about their attachments with no exam pressure and thus they could devote more time to formative assessment tasks. This does not however explain the lower level of performance by Group B, nor does it explain the low use of the online material in Year 1 by Group B. The cause of these results is unclear.

The strategy used to encourage students to use Medici could be considered to be a misuse of assessment. Certainly, students can be ‘over-assessed’ [20] but there was also an understanding that by providing the summative assessment quizzes at the end of each week online, there was likely to be collusion or circulation of the answers. Collaboration was not considered to be a poor result because students would discuss the problems before coming up with answers, but although there was anecdotal evidence of collaboration there is no evidence from this study to support that it occurred. Circulation of answers was only going to be possible if one student ‘took a bullet’ for the others by sitting the assessment and reporting the outcomes. This requires a strange version of altruism and it is equally likely that a student, having once completed the assessment and realised his or her mistakes would not allow others to gain an advantage by providing answers. Nonetheless there are certain strategies that would allow students to share the risk (and benefit) if they so desired.

Moreover, the question arises should assessment be used as a lure to encourage students to do what they should do anyway. Certainly some would argue strongly against this approach [21], just as they would argue against marks being provided for participating in tutorials or going on field trips and the point made is valid. However, there is evidence here that such a strategy is not only effective but also essential. Students prioritise tasks and they tend to prioritise to assessments. In the case of an online formative assessment task and perhaps any formative task, they chose to make it a low priority, potentially to their detriment. The year 2 group were aware that they would have to complete the cases provided online in order to perform well in the weekly assessments, however the year 1 group were also informed that the online cases linked tightly to the curriculum and it was strongly recommended that they complete the cases in order to perform well on the wards and in the end-of-attachment assessment. They chose not to do so. In all likelihood, it was the immediacy of the weekly assessment for the year 2 students, which motivated them to complete so many of the cases provided as support material. There was never any compulsion on the students to complete anything other than the 6 required 10 MCQ tests each week, yet they chose to behave differently. It is very likely that the weekly test drove the students to the online learning material, but the structure of the cases gave them an opportunity to improve their learning while they were there by engaging in optional cases. Assessment drives learning but formative assessment may not do so unless it is tightly coupled to a summative component. In the end the greatest formative assessment tools in the world are useless if nobody completes them. In addition, much time, effort and money goes into construction and production of these sorts of materials and if they are not going to be used, the money and effort might be better spent elsewhere. So a pragmatic approach may be to recognise that the reality of student life is that ‘if it doesn’t count for marks it doesn’t count at all’. This may feel a hollow victory if one believes that students should be self-motivated to complete formative assessments because they are beneficial to their learning, but it is nonetheless a victory.

Assessment is considered to have a substantial influence on student motivation [22-25] and this study supports this observation. Motivation can be regarded broadly as being either intrinsic or extrinsic. According to Deci [26] an intrinsically motivated behaviour is based on a person’s interest and enjoyment of a task with any reward being satisfaction at achieving the task or enhancing ones knowledge. Extrinsically motivated behaviour may be based on factors outside the person’s control, such as a reward for achieving the task or may also be based on internal factors, such as recognition of the value of the task. Considering the group of students from year 1, the majority presumably had little intrinsic motivation to complete the formative Medici cases and this behaviour was replicated to some degree by the behaviour of the year 2 students and the Module 0 cases. When an extrinsic motivator was applied to the students (assessment cases in each of Modules 1–6), this external regulation may have suggested to the students that the cases associated with the assessment in each module were important, and worth completing; a conscious valuing of the activity.

One of the key goals of a medical curriculum is to stimulate motivation and direction for student-centered learning. In the absence of appropriate direction, learning can be an inefficient and time-consuming process, and without suitable goals and guidelines, learners can easily drift away from areas in which they should be focussed. An important role of the teacher is to assist and guide students in their learning; to develop and define appropriate strategies for students and to help them make the most effective use of the time they have available to study. For better or worse a strong stimulus to encourage ‘learning’ is some form of assessment. Traditionally this has been in the form of summative assessment such as an end-of-course barrier examination. This method focuses students’ minds towards a single goal, but tends to foster rote learning with the inevitable “is this going to be in the exam” approach to the choice of material studied. This barrier assessment method governs student decisions on what they will attempt to learn, but is “essentially passive and does not normally have immediate impact on learning” [27]. The impact of summative assessment on the learning process for students should not be underestimated and may have a negative impact on the motivation to learn for some students [28].

A preferred stimulus for learning should be some sort of formative assessment process. The concept of formative assessment has been promoted as a means of raising the standards of achievement within the classroom, particularly in primary and secondary education [28]. Formative assessment can be defined as some form of self-assessment by the student, which will provide feedback to both teacher and student. This feedback is then used to modify teaching and learning to meet the student’s needs.

Within the clinical context formative assessment might be used to encourage appropriate professional behaviour, to foster clinical competence and to stimulate acquisition of knowledge and reasoning. Formative assessment comes in many forms and can vary from informal comments made at the end of a case presentation on a ward round to highly complex and formally structured computer-based learning tools [29,30]. Whatever the form, specialist knowledge is required to develop it and if specialist clinicians are to provide these sorts of materials for formative assessment it must be appreciated that this will be an expensive exercise and cost-effectiveness must be shown. To provide such material on a hope that it might be used is probably a waste of money and precious resources. Whilst it may appear punitive we have shown that with some gentle encouragement students will use and appreciate these materials – but when provided on an informal basis it is likely these same resources will be ignored. Considering that such encouragement – whilst punitive – has improved performance in knowledge-based assessment in this study, educators may feel this is a worthwhile approach.

## Conclusion

We have shown that a change in the strategy in the provision of materials for study-centered learning can influence the way they are used and the effect they have on acquisition of cognitive skills, particularly those requiring knowledge and understanding. In summary, the online medium is a valuable and appreciated resource, capable of providing timely formative feedback and stimulating student-centered learning. However the production of quality content is a time-consuming exercise and to ensure that the expertise required to develop such material is not wasted careful consideration must be given to the strategies employed to ensure its efficacy.

## Competing interests

The authors declare that they have no competing interests.

## Authors’ contributions

EP and PD co-developed the design of the study and were responsible for its implementation. EP wrote the first draft of the paper. PD assisted in all additional drafts. Both authors read and approved the final manuscript.

## Pre-publication history

The pre-publication history for this paper can be accessed here:

http://www.biomedcentral.com/1472-6920/14/8/prepub
